# Relationship between vitamin D and coronary artery disease in Egyptian patients

**DOI:** 10.1186/s43044-023-00419-5

**Published:** 2023-11-09

**Authors:** Magdy Algowhary, Ahmed Farouk, Heba E. M. El-Deek, Ghada Hosny, Ahmed Ahmed, Lobna A. Abdelzaher, Tahia H. Saleem

**Affiliations:** 1 Department of Cardiovascular Medicine, Assiut University Heart Hospital, Assiut University, Asyut, 71516 Egypt; 2 Department of Cardiothoracic Surgery, Assiut University Heart Hospital, Assiut University, Asyut, 71516 Egypt; 3 Department of Pathology, Faculty of Medicine, Assiut University, Asyut, 71516 Egypt; 4 Department of Public Health and Community Medicine, Faculty of Medicine, Assiut University, Asyut, 71516 Egypt; 5 Department of Pharmacology, Faculty of Medicine, Assiut University, Asyut, 71516 Egypt; 6 Department of Medical Biochemistry, Faculty of Medicine, Assiut University, Asyut, 71516 Egypt

**Keywords:** Vitamin D, Coronary artery disease, Multivessel affection, Acute myocardial infarction

## Abstract

**Background:**

Previous studies have reported conflicting results about the association of vitamin D (VD) level with coronary artery disease (CAD). We aimed to study the association of VD with atherosclerotic CAD in Egyptian individuals.

**Results:**

We prospectively enrolled 188 consecutive CAD patients with a median age of 55(50–62) years; 151(80.3%) were male. All patients were diagnosed by cardiac catheterization and were compared with 131 healthy controls. VD levels were measured in serum samples of all participants. Compared to controls, CAD patients had a significantly lower median VD level, 14.65 (9.25–21.45) versus 42.0 (32.0–53.0) ng/mL, *p* < 0.001. VD was correlated with the number of diseased coronary arteries and lipid profile (total cholesterol, low-density lipoprotein cholesterol, high-density lipoprotein cholesterol, and triglycerides, *p* < 0.001 for each). By multivariate analyses, VD was an independent predictor of CAD [OR 1.22 (95% CI 1.07–1.4), *p* = 0.003, optimal cut-off value 30 ng/mL (AUC 0.92, sensitivity 81% and specificity 81.4%), *p* < 0.001], and the number of diseased coronary arteries, *p* < 0.001, especially three-vessel disease [OR 0.83 (95% CI 0.72–0.95), *p* = 0.008].

**Conclusions:**

We have shown that low VD should be considered a non-traditional risk factor for CAD in Egyptian individuals. Low VD was correlated with coronary atherosclerosis, especially in patients with multivessel effects.

**Supplementary Information:**

The online version contains supplementary material available at 10.1186/s43044-023-00419-5.

## Background

Coronary artery disease (CAD) is a worldwide health problem. The identification of risk factors such as male sex, old age, smoking, diabetes mellitus, hypertension, dyslipidaemia, chronic kidney disease, and positive family history is important for the primary and secondary prevention of CAD. However, not all patients with CAD have traditional risk factors, suggesting other reasons for their atherosclerosis. Large cross-sectional studies with long-term follow-up of more than seven years have found that low vitamin D (VD) was associated with a high risk of cardiovascular events, myocardial infarction, and mortality [1–3]. Moreover, patients with low VD and cardiovascular disease or cerebrovascular disorder had poor outcomes, as they had increased disability and discharge to non-home facilities among hospitalization patients in the USA [4].

At the cellular level, VD receptors are expressed in many tissues of the cardiovascular system. They may control cell proliferation, apoptosis, and cell adhesion. They also downregulate proinflammatory cytokines and metalloproteinases and upregulate anti-inflammatory cytokines and inhibitors of metalloproteinases. In contrast to previous findings, the association between VD and cardiovascular disease is still controversial. Studies have shown conflicting results on the association of VD level or supplementation and CAD, endothelial function, vascular calcification, and carotid intima-media thickness [5]. Additionally, the causality of this relationship remains to be established [6].

Previous results on the effects of VD on CAD were chiefly derived from other parts of the world. This study aimed to investigate the effect of VD levels on atherosclerotic CAD and its severity in Egyptian patients who underwent diagnostic cardiac catheterization. This study was preceded by a laboratory study that consisted of immunohistochemical measurements of VD receptors in human and animal arterial tissues, which revealed deficient VD receptors in atherosclerotic specimens [7].

## Methods

### Study overview

This study was the clinical phase of a study on VD levels and atherosclerosis in Egypt. The first phase was a laboratory study that measured VD receptors in human atherosclerotic aortic specimens derived from 50 patients with myocardial infarction during surgery. That study also investigated the effects of supplementation with variable doses of VD (0.025, 0.05, and 0.075 μg/kg) on the aortas of 40 male Wistar rats. All the rats were fed a high-fat diet to induce vascular atherosclerotic processes. They were subdivided into 4 groups by their VD supplementation. The first group did not receive VD supplementation, while the rest of the 3 groups received small, medium and large doses of VD, respectively. The 4 groups were compared with a control group, 10 rats, which received a normal diet. The study found that VD receptors were low in all human aortic specimens. In animal groups, VD administration showed a dose-dependent effect on vascular VD receptors and atherosclerotic foam cell formation: Compared to the control group, the high dose VD group had marked reduction in atherosclerotic foam cell formation and the highest expression of VD receptors, while VD receptor expression and foam cell formation were also different in other VD groups (lower VD receptors and marked foam cell formation), suggesting a protective effect of VD administration [7].

### Study population

The next step in our project was to measure VD levels in a larger group of atherosclerotic patients who underwent coronary angiography. Since cardiac catheterization was the gold standard to diagnose CAD, patients were selected from those who underwent diagnostic coronary angiography. The study was a prospective study performed between March 2019 and December 2019 at our tertiary university heart hospital, before the COVID era.

The inclusion criteria were patients above 18 years old who presented with CAD (chronic stable angina pectoris, unstable angina pectoris, old myocardial infarction, acute ST-elevation and non-ST-elevation myocardial infarction). They were diagnosed by cardiac catheterization study and had a significant lesion of > 50% stenosis in any of the territories of the three epicardial coronary arteries: left main trunk, left anterior descending, left circumflex and right coronary arteries. Stenosis in the left main trunk was considered a two-vessel disease, while stenosis of the main epicardial vessel and its branches was considered a single-vessel disease. Cardiac catheterization procedures were performed through femoral or radial approaches at the operator’s discretion. Cannulation of the left and right coronary arteries was obtained by suitable catheters of the Judkins and Amplatz kinds, followed by injection of contrast media into the coronary arteries. Standard views were taken for each artery, and the worst stenosis was recorded. Standard views were taken to visualize coronary arteries in left and right views with cranial and caudal angulations to avoid missing any lesions.

History suggestive of CAD was typical chest pain or dyspnoea on exertion associated with electrocardiographic changes and ST depression > 0.5 mV in at least two contiguous leads. ST-elevation myocardial infarction was diagnosed if the patient had typical chest pain associated with ST-segment elevation (≥ 2 mm in men and ≥ 1.5 mm in women in V2-3 or 1 mm in other leads) in at least two contiguous leads or new left bundle branch block and elevated cardiac enzymes (creatine kinase, CK, creatine kinase isoenzyme, CK-MB, or troponins) [8–10]. Except in patients with posterior myocardial infarction, if the ST-segment was not persistently elevated, a diagnosis of non-ST-elevation myocardial infarction was made; otherwise, the patient was diagnosed with unstable angina pectoris (UAP) (typical chest pain changed in intensity, character, and duration, occurring after mild effort/rest, post-infarction angina or new-onset chest pain within two months without elevated myocardial enzymes) [9, 10]. Prior/old myocardial infarction was identified when pathological Q waves were present in at least two contiguous leads with or without symptoms or thinned myocardium with wall motion abnormalities due to ischaemia [11]. Detailed clinical, electrocardiographic, echocardiographic and laboratory data of all patients were recorded.

Patients were excluded if they were diagnosed with CAD by a method other than cardiac catheterization. Patients with a history suggestive of primary disorders affecting serum calcium levels, such as chronic kidney disease, bone tumours, parathyroid gland disorders, administration of VD and calcium supplementation, were excluded. Of note, chronic kidney disease patients with a glomerular filtration rate (GFR) less than 60 mL/min per 1.73 m^2^ (stages 3, 4, and 5), albuminuria, or dialysis were excluded [12, 13]. Healthy controls were individuals without a history of VD or calcium administration, or heart, parathyroid gland, or bone diseases. Additionally, persons with normal coronary arteries diagnosed by cardiac catheterization were enrolled as controls as long as previous requirements were fulfilled.

The sample size was calculated based on the results of the VITAL study, where the mean ± SD of VD was 30.8 ± 10 ng/mL [14]. Our sample size was calculated using the an independent-samples *t* test with 90% power to detect a mean difference of 5 mg between CAD patients and controls under a two-sided alpha level of 0.01. A ratio of 2:1 between patients and controls was chosen to ensure we included enough patients. The calculated sample size was 272 individuals (181 CAD patients and 91 controls). The study was approved by the ethics committee of the faculty of medicine at our university, and the IRB approval no. 17300511 was given. All participants gave written consent and the study was conducted ethically in accordance with the World Medical Association Declaration of Helsinki, 1975.

### Laboratory investigations

Five-millilitre blood samples were withdrawn from our population into plain sterile test tubes for estimation of VD and lipid profiles [total cholesterol, low-density lipoprotein (LDL) cholesterol, high-density lipoprotein (HDL) cholesterol, and triglycerides]. The tubes were centrifuged at 3000 rpm for 10 min. Sera were separated and kept at − 20 °C until the time of analysis. For measurements of total serum 25-hydroxyVD (25-OH VD), we used a total 25-OH VD EIA kit supplied by Epitope Diagnostics, Inc. San Diego, CA 92121, USA (Catalogue No: KT-715). Serum total cholesterol was quantified by an enzymatic colorimetric technique provided by Spectrum Diagnostics, (Egypt catalogue no. 230003) [15]. Serum HDL-cholesterol was estimated by the enzymatic colorimetric precipitation method supplied by Spectrum Diagnostics, (Egypt catalogue no. 266002) [16]. Serum triglycerides were estimated by the enzymatic colorimetric precipitation method supplied by Spectrum Diagnostic, (Egypt catalogue no. 314003) [17]. LDL-cholesterol was calculated from the previous data. All laboratory procedures were performed in an accredited university biochemistry laboratory.

### Statistical analysis

Continuous variables are presented as mean ± SD for normally distributed variables and median (interquartile range, IQR) for variables not normally distributed. Comparisons of means were done by using *t* tests and ANOVA. Mann–Whitney *U* and Kruskal–Wallis tests were used for variables not normally distributed. Categorical variables are presented as frequency (%). The chi-square with exact test was used for comparisons. Odds ratios (OR) were calculated using Cochran’s and Mantel–Haenszel statistics. Correlations between normal variables were measured by the Pearson test, while the Spearman test was used for nonparametric correlations. To input data into parametric tests, the data were log-transformed. Multivariate analyses were performed to identify variables independently associated with CAD, and the number of diseased coronary arteries (normal, single-, two-, and three-vessel disease) by using binary and multinomial logistic regression analyses, respectively. Linear regression analysis for VD with the forward stepwise method was also performed. Variables in the equations were sex, age, diabetes mellitus, dyslipidaemia, smoking, hypertension, family history, renal impairment (mild kidney affection, GFR ≥ 60 mL/min per 1.73 m^2^), total cholesterol, LDL-cholesterol, HDL-cholesterol, triglycerides, VD, and left ventricle ejection fraction. ROC curves were drawn to identify optimal cut-off values. All tests were performed by using SPSS package version 25 (IBM, New York, USA). All results were two-sided and *p* < 0.05 was considered significant.

## Results

### Clinical and laboratory results

A total of 188 consecutive CAD patients who underwent cardiac catheterization were included prospectively. Our CAD population had a median (IQR) age of 55 years (50–62) with a male prevalence of 80.3% and dyslipidaemia in 87.2% (Table 1). Acute coronary syndrome (ACS) was the main presentation in 131 patients (69.68%) due to acute myocardial infarction and unstable angina. ST-elevation myocardial infarction constituted 95% of patients with acute myocardial infarction. Most of the patients had a diseased left anterior descending branch (107 patients, 56.9%), and most had multivessel disease (128 patients, 68.1%). Of note, 171 patients (90.9%) underwent in-hospital revascularization by percutaneous coronary intervention for 168 patients (89.4%) and urgent coronary artery bypass surgery for 3 patients (1.6%). The other 17 patients (9%) required medical treatment or further evaluation before revascularization procedures.

**Table 1:** All patients characteristics

	Patients	Controls	*p* value
	(*n* = 188)	(*n* = 131)	
Male (%)	151 (80.3)	84 (64.1)	0.002
Age, years	55 (50–62)	52 (45–56)	< 0.001
Diabetes mellitus (%)	73 (38.8)	30 (22.9)	0.003
Hypertension (%)	84 (44.7)	63 (48.1)	0.57
Smoking (%)	74 (39.4)	9 (6.9)	< 0.001
Dyslipidaemia (%)	164 (87.2)	27 (20.6)	< 0.001
Family history (%)	0 (0)	1 (0.8)	0.41
Renal impairment (%) *	13 (6.9)	0 (0)	0.001
*Presenting symptoms (%)*			
Chest pain	163 (86.7)		
Dyspnoea	25 (13.3)		
*Clinical diagnosis (%)*			
AMI	108 (57.4)		
AP	33 (17.6)		
OMI	24 (12.8)		
UAP	23 (12.2)		
Ejection fraction (%)	47 (40–55)	55 (55–60)	< 0.001
*Coronary angiography (%)*			
Single-vessel disease	60 (31.9)		
Two-vessel disease	72 (38.3)		
Three-vessel disease	56 (29.8)		
*Culprit vessel (%)*			
LAD	107 (56.9)		
RCA	43 (22.9)		
LCX	32 (17)		
LMT	6 (3.2)		
*Intervention (%)*			
PCI	168 (89.4)		
CABG	3 (1.6)		
*Treatment (%)*			
Aspirin	188 (100)		
Ticagrelor	107 (56.9)		
Clopidogrel	73 (38.8)		
ACE-I	159 (84.6)		
ARB	5 (2.7)		
Statins	188 (100)		
Nitrates	106 (56.4)		
B-Blockers	78 (41.5)		
CA	2 (1.1)		

Data are expressed as median (IQR); frequency (%)

*ACE-I* Angiotensin converting enzyme inhibitors, *AMI* Acute myocardial infarction (acute ST-elevation and non-ST-elevation myocardial infarction), *AP* Chronic stable angina pectoris, *ARB* Angiotensin receptor blockers, *B-Blockers* Beta blockers, *CA* Calcium antagonist, *CABG* Coronary artery bypass graft, *LAD* Left anterior descending branch, *LCX* Left circumflex branch, *LMT* Left main trunk, *OMI* Old myocardial infarction, *PCI* Percutaneous coronary intervention, *RCA* Right coronary artery, *UAP* Unstable angina pectoris

*Mild kidney affection, GFR ≥ 60 mL/min per 1.73 m^2^

Compared to healthy controls, CAD patients had significantly higher median total cholesterol, LDL-cholesterol and triglycerides but lower median HDL-cholesterol and VD [VD: 14.65 (9.25–21.45) vs. 42 (32–53),* p* < 0.001] (Table 2). Moreover, VD was low in patients with multivessel disease, especially those with three-vessel disease, 12.95 (9.0–19.87), *p* < 0.001 versus controls.

**Table 2:** Vitamin D and lipid profile in CAD patients versus controls

	Patients	Controls	*p* value
	(*n* = 188)	(*n* = 131)	
Vitamin D (ng/mL)	14.65 (9.25–21.45)	42 (32–53)	< 0.001
Total cholesterol (mg/dL)	280 (230–318.25)	170 (157–187)	< 0.001
Triglyceride (mg/dL)	210 (132.25–260)	133 (119–150)	< 0.001
HDL- Cholesterol (mg/dL)	40.5 (37–48.5)	57 (50–61)	< 0.001
LDL- Cholesterol (mg/dL)	186 (158.25–237.75)	88 (70–104)	< 0.001

Data are expressed as median (IQR)

*CAD* Coronary artery disease, *HDL* High-density lipoprotein, *LDL* Low-density lipoprotein

### Measurements of VD and atherosclerotic profile

**Fig. 1:**
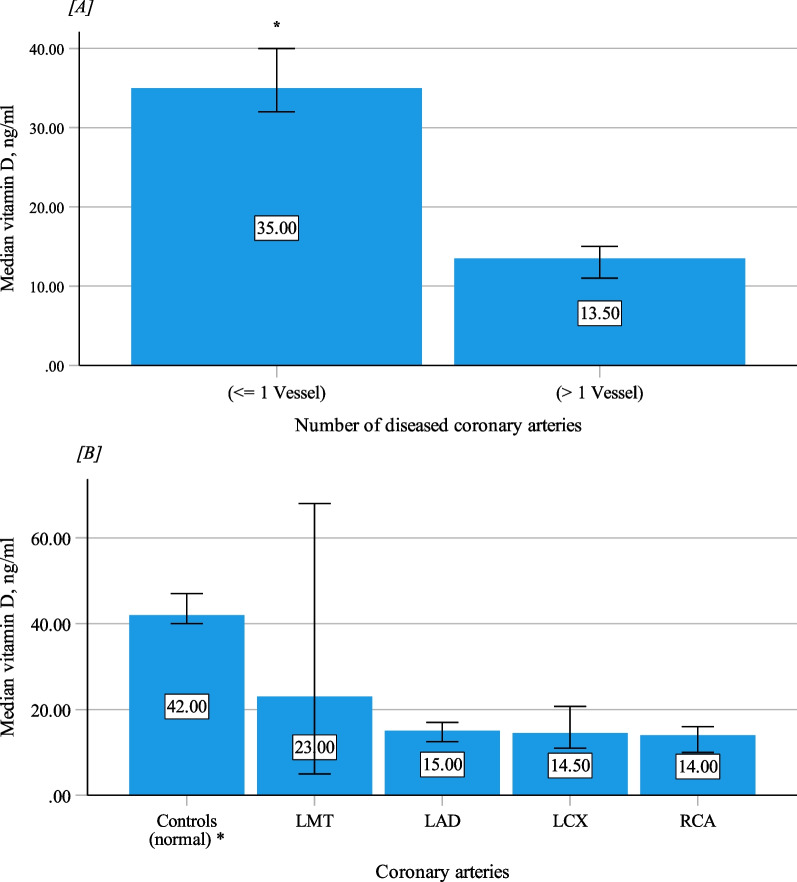
**A** Vitamin D levels according to number of diseased coronary arteries (*p < 0.001, vs. other group). **B** Vitamin D levels according to diseased coronary arteries and controls (*p < 0.001, vs. other groups). (Abbreviations: LAD: Left anterior descending artery, LCX: Left circumflex artery, LMT: Left main trunk, RCA: Right coronary artery)

Figure 1A and B show the measurements of VD according to the number of diseased coronary arteries and the site of atherosclerotic vessels. VD measurements are comparable regardless of the location of the atherosclerotic plaque. Nevertheless, the lowest measurement of median VD was recorded in patients who had right coronary vessel disease, 14.0 (9.0–18.0), *p* < 0.001 versus controls.

**Fig. 2:**
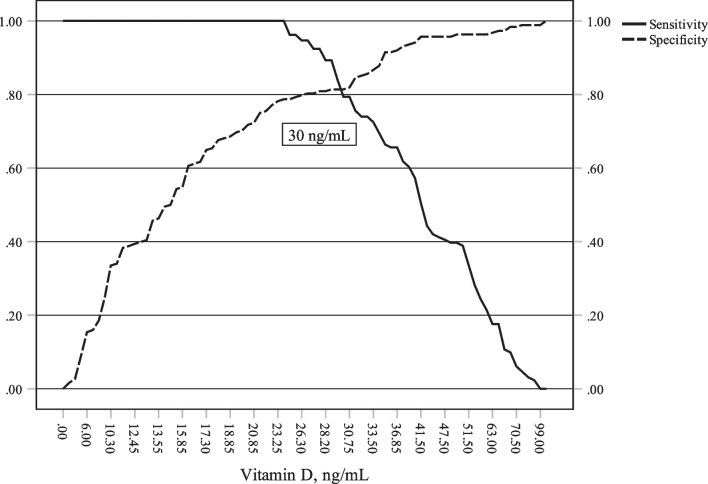
Cut-off value of vitamin D to detect coronary artery disease

Table 3 shows the independent predictors of CAD in our population produced by multivariate analysis. The model classified 98.7% of the population correctly. Low VD independently predicted CAD [OR (95% CI) 1.22 (1.07–1.40), *p* = 0.003]. Its cut-off value was 30 ng/dL, which had a sensitivity of 81% and a specificity of 81.4%, with an area under the curve of 0.92 (*p* < 0.001) (Fig. 2). Based on the cut-off value, our study population could be classified into two groups: low VD (< 30 ng/mL), and high VD (≥ 30 ng/mL). Individuals in the low VD group were significantly older and more often had diabetes mellitus, smoking, dyslipidaemia, renal impairment and male prevalence than those in the high VD group (Table 4). The median measurements of total cholesterol, LDL-cholesterol, and triglycerides were significantly higher in the low VD group than in the high VD group (*p* < 0.001 for each), while the protective HDL-cholesterol was significantly lower (*p* < 0.001). Importantly, individuals who had low VD more frequently had multivessel disease [60.6%, OR 4.43 (2.87–6.84), *p* < 0.001], and more often had ACS [57.3%, OR 2.84 (1.99–4.05), *p* < 0.001], especially acute myocardial infarction [47.8% vs. 15.8%, respectively, *p* < 0.001], than individuals who had high VD.

**Table 3:** Multivariate analyses of CAD and number of diseased vessels

	OR (95% CI)	*p* value
CAD*		
Vitamin D	1.22 (1.07–1.40)	0.003
Total cholesterol	0.94 (0.9–0.98)	0.003
HDL-C	1.18 (1.05–1.33)	0.006
LDL-C	0.93 (0.88–0.98)	0.006
Smoking	0.033 (0.002–0.52)	0.015
EF	1.43 (1.08–1.88)	0.012
Number of diseased vessels**		
Vitamin D	18.9	< 0.001
EF	18.6	< 0.001
LDL-C	12.7	0.005
Diabetes mellitus	9.5	0.024
Total cholesterol	7.9	0.049
Smoking	7.1	0.068

*CAD* Coronary artery disease, *CI* Confidence interval, *EF* Left ventricle ejection fraction, *HDL-C* High-density lipoprotein cholesterol, *OR* Odds ratio, *LDL-C* Low-density lipoprotein cholesterol, *TG* Triglycerides

*Results of binary logistic regression analysis

**Results of Multinomial logistic regression analysis

**Table 4:** Comparison between low and high vitamin D groups

		Low vitamin D	High vitamin D	*p* value	OR (95% CI)	*p* value *
		(< 30 ng/mL)	(≥ 30 ng/mL)			
		(*n* = 180)	(*n* = 139)			
CAD (%)		153 (85)	35 (25.2)	< 0.001	16.8 (9.6–29.5)	< 0.001
Male (%)		141 (78.3)	94 (67.6)	0.04	1.73 (1.05–2.86)	0.032
Age (years)		55 (50–60)	52 (46–58)	0.006		
Diabetes mellitus (%)		69 (38.3)	34 (24.5)	0.011	1.92 (1.2–3.1)	0.009
Hypertension (%)		82 (45.6)	65 (46.8)	0.91	0.95 (0.6–1.5)	0.83
Smoking (%)		62 (34.4)	21 (15.1)	< 0.001	2.95 (1.7–5.2)	< 0.001
Dyslipidaemia (%)		140 (77.8)	51 (36.7)	< 0.001	6.04 (3.7–9.9)	< 0.001
Family history (%)		0	1 (0.7)	0.44		
Renal impairment (%)		11 (6.1)	2 (1.4)	0.05	4.5 (0.97–20.5)	0.054
EF (%)		50 (42.8–55)	55 (55–60)	< 0.001		
*Clinical diagnosis (%)*				< 0.001		
All population	ACS	103 (57.3)	28 (20.1)		2.84 (1.99–4.05)	< 0.001
	Non-ACS	77 (42.8)	111 (79.9)			
CAD patients	ACS	103 (78.6) **	28 (21.4)	0.16	1.94 (0.79–4.75)	0.16
	Non-ACS	50 (87.7) **	7 (12.3)			
*lipid profile (mg/dL)*						
	TC	267 (200–310)	180 (160–200)	< 0.001		
	TG	200 (130–250)	133 (119–160)	< 0.001		
	HDL-C	42 (38–50)	52 (47–60)	< 0.001		
	LDL-C	174 (125.8–225.3)	100 (80–116)	< 0.001		
*Number of diseased coronary vessels*				< 0.001		
	> 1	109 (60.6)	19 (13.7)		4.43 (2.87–6.84)	< 0.001
	≤ 1	71 (39.4)	120 (86.3)			
*Diseased coronary vessel (%)*				0.085		
	LAD	85 (47.2)	22 (15.8)			
	RCA	39 (21.7)	4 (2.9)			
	LCX	26 (14.4)	6 (4.3)			
	LMT	3 (1.7)	3 (2.2)			

Data are expressed median (IQR); frequency (%)

*ACS* Acute coronary syndrome (acute myocardial infarction and unstable angina pectoris), *CAD* Coronary artery disease, *CI* Confidence interval, *EF* Left ventricle ejection fraction, *HDL-C* High-density lipoprotein cholesterol, *LAD* Left anterior descending artery, *LCX* Left circumflex artery, *LDL-C* Low-density lipoprotein cholesterol, *LMT* Left main trunk, *OMI* Old myocardial infarction, *OR* Odds ratio, *RCA* Right coronary artery, *TC* Total cholesterol, *TG* Triglycerides

*Significance of OR

**p < 0.001 (versus controls)

### Association of VD with CAD and its severity

Low VD was correlated with the number of diseased coronary arteries (*r* = − 0.7), total cholesterol (*r* = − 0.6), LDL-cholesterol (*r* = − 0.6), HDL-cholesterol (*r* = 0.5), triglycerides (*r* = − 0.4), left ventricle ejection fraction (*r* = 0.4), smoking habit (*r* = − 0.3), and age (*r* = − 0.2) (*p* < 0.001 for each correlation) (Additional file 1: Fig. 3). Additionally, significant but weaker correlations were detected with diabetes mellitus (*r* = − 0.15, *p* = 0.007), renal impairment (*r* = − 0.15, *p* = 0.008), and sex (*r* = 0.13, *p* = 0.019).

By multivariate analyses, low VD measurement was an independent predictor of not only CAD (*p* = 0.003) but also the number of diseased coronary arteries (*p* < 0.001) (Table 3). Of note, the degree of significance was highest with three-vessel involvement [controls, OR 1.22 (95% CI 1.05–1.42), *p* = 0.01; single-vessel disease, OR 0.84 (95% CI 0.73–0.96), *p* = 0.011; two-vessel disease, OR 0.84 (95% CI 0.73–0.96), *p* = 0.012; and three-vessel disease, OR 0.83 (95% CI 0.72–0.95), *p* = 0.008]. These results were confirmed by a separate linear regression analysis on VD concentrations, which revealed a significant association with CAD [OR 0.86 (95% CI 0.57–1.14), *p* < 0.001] and the number of diseased vessels [OR − 0.15 (95% CI − 0.27 to − 0.03), *p* = 0.015].

## Discussion

The chief findings of this study are that low VD concentrations in the blood were significantly associated with CAD and its severity in Egyptian patients. This prospective study found that low VD levels independently predicted atherosclerotic coronary artery lesions in ischaemic patients. Moreover, it was an independent predictor of multivessel coronary affection diagnosed by cardiac catheterization. The study complemented the laboratory stage of this work that previously revealed the existence of low VD receptor expression in human and animal atherosclerotic arterial strips [7]. Taken together, our findings of low VD levels in the blood of CAD patients and a high prevalence of low VD receptors in atherosclerotic arteries confirmed the significant role of VD in the pathogenesis of atherosclerosis. The most important aspect of the study was its prospective design, which avoided the problems associated with the retrospective collection of data, and the inclusion of a large number of Egyptian patients who underwent cardiac catheterization.

The aetiology of cardiovascular disease is not completely understood. Geographic factors, including place of residence, nutrition, and exposure to sun, may be related to cardiovascular disorders, including atherosclerotic coronary artery disease. These environmental data may influence the exposure of humans to ultraviolet rays and consequently their VD levels. The median level of VD in CAD patients was significantly lower than that in controls [14.65 (9.25–21.45) vs. 42.0 (32–53), *p* < 0.001]. It acted concurrently with the atherosclerotic lipid profile (high total cholesterol, LDL-cholesterol and triglycerides and low HDL-cholesterol) on the coronary arteries to cause atherosclerosis. Zittermann et al. illustrated how low 25-OH VD levels could result in atherosclerotic changes [18]. Several pathways might be responsible for the pathogenesis of atherosclerosis. 1,25 DihydroxyVD (a precursor of renal conversion of 25-OH VD by 1α-hydroxylase) inhibits the proliferation of vascular smooth muscle cells by acute influx of calcium into the cells. Thus, low VD results in the proliferation of smooth muscle cells, causing thickening of the vascular endothelium. It is also responsible for the low production of matrix G1α protein from vascular smooth muscle cells, triggering massive vascular calcification. Moreover, serum parathyroid hormone might be increased as a compensatory mechanism to raise calcium levels eliciting calcium and phosphate deposition in vessel walls and more insulin resistance. Thickening of the vessel wall due to the proliferation of smooth muscle cells and calcification could encroach into the coronary lumen markedly resulting in myocardial ischaemia and even infarction.

Atherosclerosis is a systemic disease characterized by chronic inflammation with the accumulation of lipids, smooth muscle cell proliferation, cell apoptosis, necrosis, fibrosis, and local inflammation. Endothelial inflammation releases proinflammatory cytokines and adhesive molecules such as interleukin 6 (IL-6), monocyte chemotactic protein 1 (MCP-1), intercellular adhesion molecule 1 (ICAM-1) and vascular cell adhesion molecule 1 (VCAM-1), initiating monocyte adhesion and infiltration of the vessel wall. Consequently, proinflammatory cytokines such as tumour necrosis factor-α (TNF-α) and interferon-γ (IFN-γ) will be released in the vascular environment. Monocyte-derived macrophages engulf oxidized low-density lipoprotein, forming foam cells and fatty streaks and resulting in plaque formation in coronary arteries. The process is tightly regulated by cytokines [19]. Low VD, as noticed in our patients, may be associated with increased levels of proinflammatory cytokines such as TNF-α and IL-6 and decreased levels of anti-inflammatory cytokines such as interleukin-10 (IL-10), enhancing atherosclerotic plaque formation. This is accompanied by endothelial dysfunction [20] and increased renin-angiotensin system expression, aggravating atherosclerotic processes, hypertension and cardiovascular risk [6, 21]. Although the association between low VD and atherosclerosis denotes a causative link, it might be a consequence of atherosclerosis [6]. On the other hand, the high vitamin D group in our population had a significantly higher level of HDL-cholesterol than the low vitamin D group. Recently, HDL-cholesterol has been found to prevent inflammation and atherosclerotic processes [22].

We diagnosed CAD solely by coronary angiography, which is currently considered the gold-standard method. Other modalities used to diagnose CAD and the number of diseased coronary arteries, such as electrocardiographic examination, exercise stress test, stress test and computed tomography yield higher rates of false results. Nevertheless, similar studies that relied on cardiac catheterization to diagnose coronary atherosclerosis and to measure VD in patients with CAD were reported [20, 23]. It is worth mentioning that coronary revascularization depends chiefly on cardiac catheterization [24].

Our study revealed a significant association between low VD and atherosclerotic CAD. Low VD was an independent predictor of atherosclerotic CAD and was correlated with traditional coronary risk factors, although the correlations were not very strong. A large cross-sectional study showed that patients with a high VD level, ≥ 30 ng/mL, had a more favourable lipid profile than patients with a low VD level, < 20 ng/mL [25], so low VD might be considered a cardiovascular risk factor [26]. More recently, measurements of VD in patients with ST-elevation myocardial infarction were consistent with our study [23]. Of note, most of our patients who presented with acute myocardial infarction (57.4%) had ST-elevation myocardial infarction. Recently, VD receptors were discovered in more than 30 cell types [27]; therefore, they carry out different functions rather than participating in mineral homeostasis alone [28]. VD receptors in the blood vessels might be responsible for the integrity of the vascular wall. Few VD receptors are expressed in the endothelial and smooth muscle cells of atherosclerotic and preatherosclerotic lesions [5, 7, 29, 30].

Most [103 (57.3%)] of our patients low VD (< 30 ng/mL) had ACS, including unstable angina, ST-elevation myocardial infarction and non-ST-elevation myocardial infarction. The odds of low VD in patients with ACS were higher than they were in patients with high VD [OR 2.84 (95% CI 1.99–4.05), *p* < 0.001]. The rupture of vulnerable plaques was thought to be responsible for most of these events. Vulnerable plaques are characterized by rich lipid content, a thin fibrous cap and high inflammatory activity. Although the molecular function of VD signalling is still not clear [28], VD was shown to reduce the accumulation of cholesterol in macrophages and decrease LDL-cholesterol uptake in atheromas [31]. These favourable effects were associated with inhibition of platelet aggregation and reduction of thrombogenic activity through modulation of thrombomodulin and tissue factor expression in monocytes [32]. Thus, low VD triggers cholesterol accumulation and enhances thrombogenic activity. Additionally, plaque destabilization was boosted through the increase in expression of matrix metalloproteinase (MMP)-2 and MMP-9 which was evident in blood samples derived from male patients with acute myocardial infarction. Therefore, plaques become more vulnerable to rupture, causing ACS [33, 34]. These effects of low VD levels also explain the findings in our study and other prospective studies [2]. Giovannucci et al. demonstrated that the risk of myocardial infarction was significantly higher in men who had VD ≤ 15 ng/mL than those with sufficient VD (≥ 30 ng/mL) at ten-year follow-up [relative risk 2.42 (95% CI 1.53–3.84), *p* < 0.001] [2]. Of note, our CAD patients had a similar median level of VD, 14.65 ng/mL, explaining why acute myocardial infarction predominated. The study centre was a referral centre for primary coronary intervention, which partly explains why acute myocardial infarction was common in our population. We found a cut-off value of VD at 30 ng/mL, below which atherosclerotic coronary artery disease was more likely to occur. A similar level was reported before [11, 21, 25].

Multivessel disease of coronary arteries would carry an increased risk of future cardiovascular events in CAD patients. Two- and three-vessel disease were prevalent in the majority of patients with low VD, 109 patients (60.6%). The OR of having more than single-vessel disease in the population with low VD versus those with high VD was 4.43 (95% CI 2.87–6.84, *p* < 0.001). Taking into consideration that other variables, such as diabetes mellitus, left ventricular ejection fraction, LDL-cholesterol, total cholesterol and smoking, were important, a low VD level was an independent predictor of multivessel disease of coronary arteries in our population. However, in peripheral arterial disease, conflicting results of VD levels have been reported [35, 36]. Nevertheless, multivessel disease was seen more frequently in patients with low VD than in those with high VD levels [20]. More confirmatory studies identified a significant association between low VD and the severity of coronary artery disease as assessed by the SYNTAX score [37, 38]. These studies proved that low VD levels were significantly associated with high SYNTAX score (> 22) but not in patients undergoing surgery [39].

To summarize, the study showed that VD was essential for the integrity of coronary arteries, while its reduction was associated with the occurrence of atherosclerotic CAD, especially multivessel disease and possibly ACS. On the other hand, based on the results of the VITAL and WHI studies, which demonstrated that VD supplementation failed to prevent cancer, cardiovascular events [14], mortality [40], and even bone fracture [41, 42], VD supplementation should not be prescribed for primary prevention of CAD [43–45], but it would be beneficial in treating CAD patients, especially those with multivessel disease and ACS. Hence, prospective trials would be helpful to demonstrate the effect of VD supplementation in treating CAD patients, especially those with multivessel disease and ACS.

## Study limitations

Cytokines and endothelin were not measured. Comparing VD levels among diseased coronary arteries (left min trunk, left anterior descending, left circumflex, and right coronary) yielded no significant differences because too few patients were in each group. The study was not powered to investigate the association of VD and ACS.

## Conclusions

VD level was significantly lower than normal in Egyptian patients with CAD. In addition to the traditional cardiovascular risk factors, low VD was associated with coronary artery atherosclerosis and its severity. Prospective trials to demonstrate the effect of VD supplementation to treat CAD patients are encouraged, especially in those with multivessel disease and acute coronary syndrome.

### Supplementary Information

**Additional file 1: Fig. 3** **A** Correlation between log vitamin D and log total cholesterol (*r *= − 0.55, *p *< 0.001). **B** Correlation between log vitamin D and log LDL-C (*r *= − 0.504, *p *< 0.001). **C** Correlation between log vitamin D and log HDL-C (*r *= 0.38, *p *< 0.001). **D** Correlation between log vitamin D and log TG (*r *= − 0.28, *p *< 0.001). (Abbreviations: HDL-C: High-density lipoprotein, LDL-C: Low-density lipoprotein cholesterol, TG: Triglycerides).

## Data Availability

The manuscript data is available on request to the corresponding author.

## Abbreviations

ACS: Acute coronary syndrome
CAD: Coronary artery disease
CK-MB: Creatine kinase myocardial band
CK: Creatine kinase
HDL: High density lipoprotein
ICAM-1: Intercellular adhesion molecule-1
IFN-c: Interferon-c
IL-10: Interleukin-10
IL-6: Interleukin-6
IQR: Interquartile range
GFR: Glomerular filtration rate
LDL: Low density lipoprotein
MCP-1: Monocyte chemotactic protein-1
MMP-2: Matrix metalloproteinase-2
MMP-9: Matrix metalloproteinase-9
OH VD: Hydroxyvitamin D
OR: Odds ratio
SYNTAX: The Synergy between Percutaneous Coronary Intervention with Taxus and Cardiac Surgery
TNF-a: Tumour necrosis factor-a
UAP: Unstable angina pectoris
VCAM-1: Vascular cell adhesion molecule-1
VD: Vitamin D
VITAL: Vitamin D and omega-3 trial
WHI trial: Women’s Health Initiative calcium-vitamin D randomized controlled trial
