# Supplementary figures and images for: Relationship between vitamin D and coronary artery disease in Egyptian patients
Source: Egypt Heart J. 2023 Nov 9;75:92. doi: 10.1186/s43044-023-00419-5 (PMC10635996; doi:10.1186/s43044-023-00419-5)

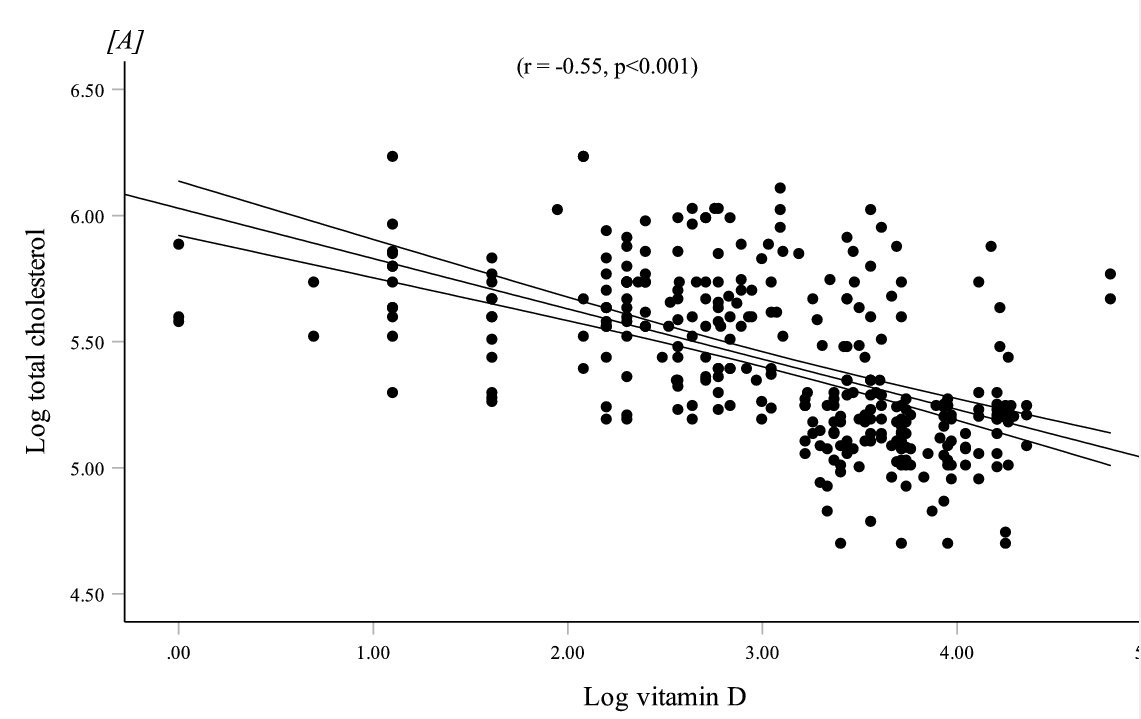


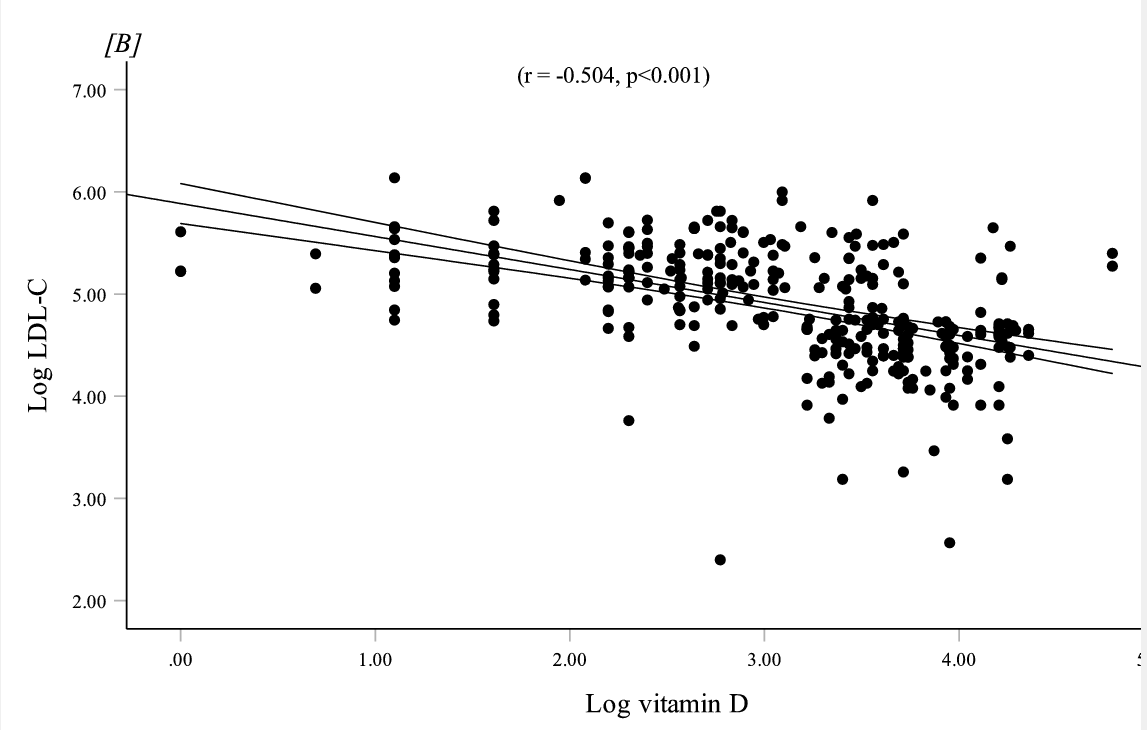


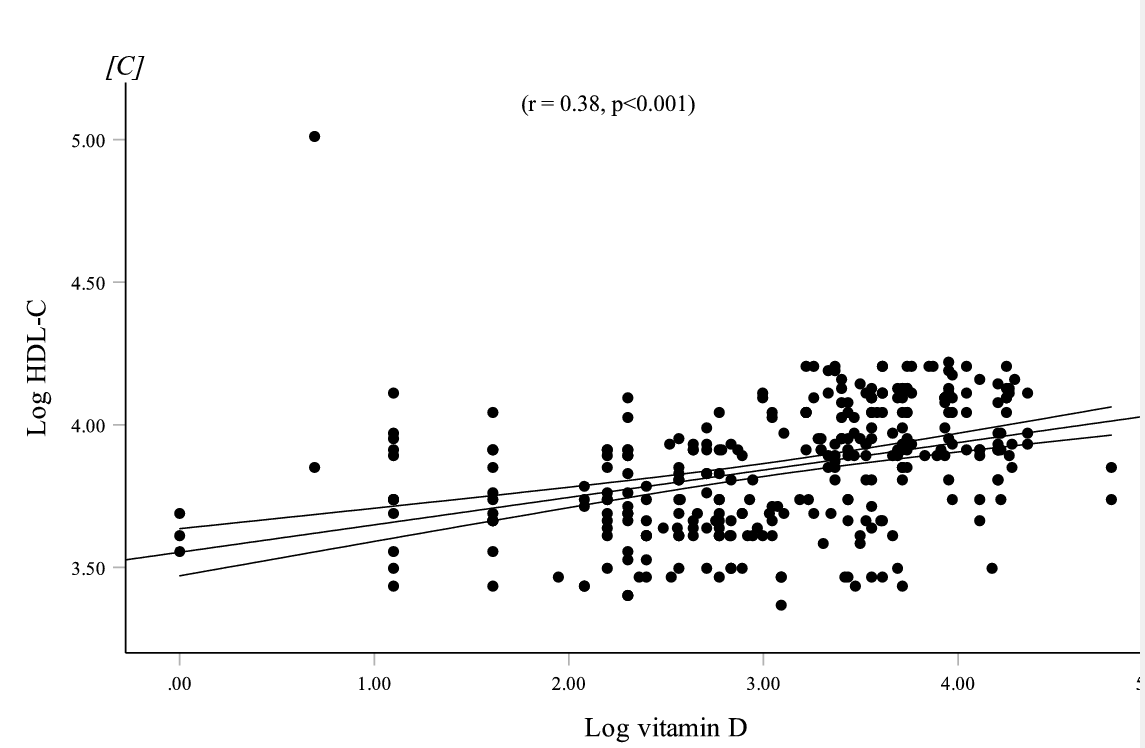


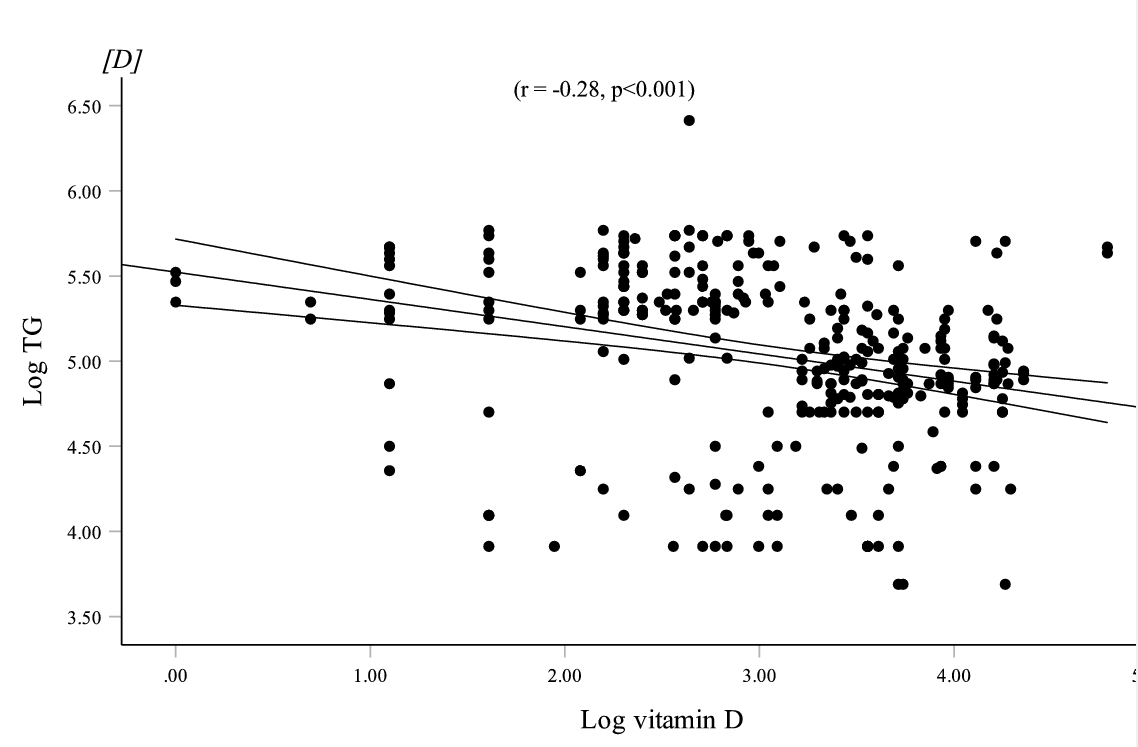

Supplement: Supplementary file 1 — Additional file 1: Fig. 3 A Correlation between log vitamin D and log total cholesterol (r = − 0.55, p < 0.001). B Correlation between log vitamin D and log LDL-C (r = − 0.504, p < 0.001). C Correlation between log vitamin D and log HDL-C (r = 0.38, p < 0.001). D Correlation between log vitamin D and log TG (r = − 0.28, p < 0.001). (Abbreviations: HDL-C: High-density lipoprotein, LDL-C: Low-density lipoprotein cholesterol, TG: Triglycerides). [file 43044_2023_419_MOESM1_ESM.docx]
